# Impacts of Color Coding on Programming Learning in Multimedia Learning: Moving Toward a Multimodal Methodology

**DOI:** 10.3389/fpsyg.2021.773328

**Published:** 2021-12-03

**Authors:** Yang Liu, Weifeng Ma, Xiang Guo, Xuefen Lin, Chennan Wu, Tianshui Zhu

**Affiliations:** School of Information and Electronic Engineering, Zhejiang University of Science and Technology, Hangzhou, China

**Keywords:** color coding, EEG, eye-tracking, programming learning, multimedia learning

## Abstract

In the present study, we tested the effectiveness of color coding on the programming learning of students who were learning from video lectures. Effectiveness was measured using multimodal physiological measures, combining eye tracking and electroencephalography (EEG). Using a between-subjects design, 42 university students were randomly assigned to two video lecture conditions (color-coded vs. grayscale). The participants’ eye tracking and EEG signals were recorded while watching the assigned video, and their learning performance was subsequently assessed. The results showed that the color-coded design was more beneficial than the grayscale design, as indicated by smaller pupil diameter, shorter fixation duration, higher EEG theta and alpha band power, lower EEG cognitive load, and better learning performance. The present findings have practical implications for designing slide-based programming learning video lectures; slides should highlight the format of the program code using color coding.

## Introduction

Each object has its own color, be it a color such as red, green, or blue, or be it colorless (i.e., white, black, or gray). A considerable amount of scientific research on color psychology has been conducted on all aspects of color. The theoretical basis of color and psychological functioning is based on the physiological model proposed by Goldstein (1942). Goldstein discussed that color naturally causes physiological reactions that manifest in emotional experience, cognitive focus, and motor behavior.

Color carries a psychologically relevant meaning, and the colors seen influence psychological functioning (Elliot and Maier, 2012). The first study on this issue (Schauss, 1979) suggested that pink reduced physical strength and aggression. Black (Frank and Gilovich, 1988) is associated with evil, death, and other negative concepts. Red is related to happiness and facilitates non-systematic cognitive processing (Soldat et al., 1997). Therefore, color can be regarded as a type of non-content visual stimulus.

According to perceptual theory, stimuli with distinguishing features, such as color, are conducive to visual search (Treisman and Gelade, 1980; Itti and Koch, 2000). Color coding impacts learners’ cognitive processing as material-oriented interventions in multimedia learning. The salience of color can attract learners’ attention and guide it toward the relevant visual information that designers want learners to process (Hillstrom and Chai, 2006; Jamet et al., 2008). From the perspective of perceptual theory, this is a bottom-up processing method that affects the learning process and learning effect through the visual design of multimedia.

In his Cambridge Handbook of Multimedia Learning, Mayer classifies color as visual signaling (Mayer, 2005a). The signaling principle, also known as the cueing principle, points out that people learn more deeply from multimedia materials when signals are added to guide their attention to the relevant elements of the material or highlight the organization of the material (Mayer, 2005a; de Koning et al., 2009).

When color coding is added to highlight the organization of learning materials, learners can obtain more information from multimedia messages. In the process of multimedia learning, learners must integrate the information conveyed through external representations into coherent mental representations. However, there is evidence that learners often need instructional support to identify correspondence and establish relationships between them (e.g., Seufert, 2003; Seufert and Brünken, 2006). Color coding is the non-lexical information added to learning materials that can attract learners’ attention and promote the selection, organization, and integration of instructional elements (de Koning et al., 2009). Many studies have shown that color coding in multimedia materials facilitates learning (Jeung et al., 1997; Kalyuga et al., 1999; de Koning et al., 2007; Ozcelik et al., 2009; Jamet, 2014; Richter et al., 2016).

Many researchers have explained from the perspective of cognitive load theory that color cues can reduce learners’ overall cognitive load and avoid overload, resulting in improved academic performance (Sweller, 1988; Paas et al., 2003).

Subjective measures are the most common methods used to assess cognitive processing (Mutlu-Bayraktar et al., 2019). Subjective self-reports on personal feelings can provide valuable information, but validity and corroboration problems arise (Bethel et al., 2007). Participants may not answer exactly how they feel, but in the way they believe others will answer. Furthermore, color is an implicit affective cue that seems to influence psychological functioning without people’s awareness (Friedman and Förster, 2010). Thus, physiological signals help to better understand the potential responses of participants during observations.

Eye tracking data can offer valuable information about learners’ cognitive processes. As a cognitive processing assessment method, it helps reveal information about the underlying cognitive processes during multimedia learning (Just and Carpenter, 1976; Antonenko et al., 2010; van Gog and Scheiter, 2010). Many studies have incorporated eye tracking measures to investigate perceptual processes as indicators of cognitive activity (e.g., Hyönä, 2010; Jarodzka et al., 2010; Park et al., 2015a,b). For instance, pupil diameter has been shown to be a sensitive measure of mental demands. Usually, the pupil dilates to meet increased task processing demands (van der Wel and van Steenbergen, 2018; Scharinger et al., 2020). Furthermore, fixation duration is related to the continuous psychological processes associated with present information (Just and Carpenter, 1976). Total fixation time is considered to be a sign of total cognitive processing engaged with fixation information (Just and Carpenter, 1980; Rayner, 1998). Therefore, fixation duration is a very useful measure of cognitive load (Holmqvist et al., 2017).

Electroencephalography (EEG) can non-invasively assess brain activity in an authentic environment. It measures the electrical activity generated by the brain through electrodes placed on the scalp. Changes in the power of neural oscillations measured with EEG are also a credible method for examining cognitive processes in multimedia learning (Antonenko and Niederhauser, 2010). EEG signals are voltage signals generated by neural activities, and these neural activities change with cognitive processes such as learning (Lin and Kao, 2018). Moreover, EEG power reflects the capacity or performance of cortical information processing. Many prior studies have used EEG to detect, estimate, or predict human brain activities (Gevins and Smith, 2003; Galán and Beal, 2012; Yoshida et al., 2014). These measurements vary with different levels of cognitive stimulation (Berka et al., 2004), which makes it a credible choice for measuring and continuously assessing cognitive load in a learning environment (Klimesch, 1999; Anderson and Bratman, 2008).

The alpha and theta changes in rhythms reflect what is happening in a subject’s information processing, even if the subject is not aware of such changes or cannot express them in words (Basar, 1999; Klimesch et al., 2005). A number of researchers have shown that alpha and theta activities are the most relevant measure of task difficulty or cognitive load amidst various task demands (Penfield and Jasper, 1954; Gale and Christie, 1987; Sterman et al., 1994; Gevins et al., 1997). Beta waves have also been demonstrated to be related to perception and cognition (Singer, 1993; Haenschel et al., 2000; Wrobel, 2000). Increased beta waves have been correlated with anxious thinking.

Electroencephalography signals are non-stationary, dynamic, and non-linear time series. Many researchers have used non-linear parametric measures to quantify EEG data (Gribkov and Gribkova, 2000). The degree of chaos in time series data can be measured using wavelet entropy (Rosso et al., 2001), sample entropy, and approximate entropy (Richman and Randall, 2000). Entropy is an accurate measure of complexity (Pincus, 1995; Chen et al., 2009), i.e., higher entropy refers to higher complexity (Parbat and Chakraborty, 2021).

Color acts as a non-conscious prime that affects psychological function in subtle ways (Bargh, 1990). Meanwhile, EEG has a high temporal resolution and has been proven to accurately reflect subtle changes that can be distinguished and quantified in a second-by-second time frame. Therefore, EEG can provide an index of the utilization of resources for the color coding effect on learners.

In the present study, we sought to determine the color coding effect on programming learning in multimedia learning. Based on the above analysis, we hypothesized that color coding is beneficial for learning. We recorded eye tracking and EEG data simultaneously to provide further insights on assessing cognitive processing. These recordings stemmed from both the central (i.e., brain) and autonomic (i.e., pupillary response) nervous systems. We focused on measuring the EEG theta, alpha, and beta frequency band power; pupil dilation; and fixation duration – all of which are specific indications of cognitive processing needs.

## Method

### Participants

The participants comprised 42 graduate students recruited from the Zhejiang University of Science and Technology in China. There were 26 male and 16 female participants, all of whom were over 18 years old (*M* = 20.81, SD = 1.13).

The participants were selected from those who did not take the “Object-oriented Python Programming” course in order to ensure that the participants did not have any prior knowledge of the learning material. However, all participants had taken C/C++ courses to ensure that they had the necessary programming foundation. In addition, their C/C++ course scores (with a maximum possible score of 100) were used to ensure that the participants had similar prior knowledge of the learning material.

All participants had normal or corrected-to-normal vision and hearing. They signed an informed consent form before the experiment and received a small gift at the end of the study to thank them for their time and effort.

### Materials

Two different versions of video lectures were used in the present study, which differed only with regard to the color of the presented text. All other features were maintained at constant values.

**FIGURE 1:**
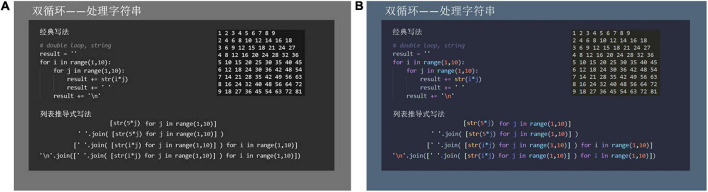
Two slide-based video lectures in **(A)** grayscale design and **(B)** color-coded design.

The learning materials are shown in Figure 1. Each video lecture included the same PowerPoint presentation, accompanied by a lecture given by a professor who is proficient in teaching the topic. As illustrated in Figure 1A, the grayscale design was achromatic. Figure 1B shows the color-coded design which used the “Palenight Theme” to highlight code format, which is widely utilized in programming. The video lectures were identical in speed, sound, and light. The materials were not self-paced and did not allow learners to start, stop, or replay short sequences. Each video lecture lasted approximately 5 min and introduced the topic of “List Expression in Python” in Chinese.

### Eye Tracking and Electroencephalography Recording

A Tobii T120 Eye Tracker device was used to collect data regarding the participants’ eye movements. The data rate of Tobii T120 for tracking was 120 Hz. Participants completed a 9-point calibration and validation procedure before watching the video lecture. They sat in front of a 17-inch monitor to view the lectures at a distance of 60 cm.

Electroencephalography was recorded at 15 electrode sites (Fp1, Fp2, F7, F3, Fz, F4, F8, T7, Cz, T8, P7, Pz, P8, O1, O2), which were positioned according to the international 10–20 system (Jasper, 1958). CPz served as a reference during recording. Data were referenced to the average of all electrodes and the ground electrode was located at the AFz. The conductive gel was inserted into each electrode with a blunt needle syringe to reduce the impedance to <5 kΩ.

### Measurements

#### Perceptions of Task Difficulty

The participants completed a nine-point Likert-type (1 = strongly easy, 9 = strongly difficult) survey on their perceptions of task difficulty, which asked the question, “How easy or difficult was the material to understand?” (Kalyuga et al., 2000). This item has been proven to be a reliable questionnaire for measuring the extraneous cognitive load experienced by learners in the learning process (Tuovinen and Paas, 2004; Kalyuga and Sweller, 2005).

#### Individual Interest in Programming Learning

To avoid the influence of individual interest on the experimental results, the direct measurement method was used to measure the subjects’ interest in program learning. All participants were also given a nine-point Likert-type (1 = strongly uninterested, 9 = strongly interested) measure of their individual interest in programming learning, which asked, “How much you did like computer programming learning?”

#### Learning Performance Test

A learning performance test was developed to measure the acquisition of knowledge presented in the video lecture. It measured the learners’ understanding of key concepts in the learning materials and their ability to apply the concepts to solve problems. The test consisted of ten items and the participants received one point for each correct answer. The total possible learning performance score was 10.

### Procedure

**FIGURE 2:**
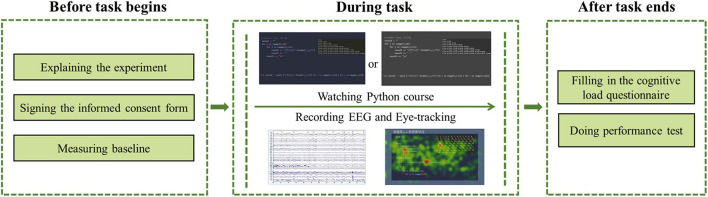
Experimental procedure. The experiment used a between-subjects design.

As illustrated in Figure 2, the participants were randomly assigned to one of the two following video lecture conditions: the grayscale design condition (*n* = 21) and the color-coded design condition (*n* = 21). The procedure was conducted through computer programs and paper-and-pencil questionnaires. Each subject was individually tested in a laboratory setting for approximately 40 min. Prior to the task, each participant learned about the experimental procedure and signed an informed consent form. The participants then closed their eyes for 3 min so we could take a baseline measurement. Thereafter, the participants viewed one of the video lectures. Participants’ EEGs and eye tracking data were recorded synchronously during the entire experiment. Finally, the participants completed the cognitive load questionnaire and learning performance test immediately after viewing the assigned lecture.

### Data Preprocessing and Analysis

The EEG data analysis was performed using MATLAB. Four data points were deleted for low-signal quality. Therefore, the color-coded design group included 19 data points and the grayscale design group included 19 data points.

First, the DC components were removed at 1 Hz using a finite impulse response (FIR) high-pass filter. Then, the other artifact noises at high frequencies were removed using an FIR low-pass filter at 50 Hz. Subsequently, the EEG data were referenced by subtracting the average of all collected electrodes from each individual electrode. An independent component analysis was also conducted to remove electrooculography (EOG) and eye artifacts (Jung et al., 2000; Delorme et al., 2007). Finally, the Hilbert–Huang transform (HHT) technique was used to calculate the power spectrum of each EEG epoch (4 s) with a frequency resolution of 0.1 Hz (Huang et al., 1998).

In the present study, we applied both the linear method (i.e., average power) and non-linear method (i.e., spectral entropy) on EEG data to evaluate the participants’ cognitive processing.

The power of five standard EEG rhythms, i.e., delta (0.5–4 Hz), theta (5–8 Hz), alpha (9–12 Hz), beta (13–32 Hz), and gamma (33–50 Hz), were computed by averaging the spectral powers in the corresponding frequency bands (Sghirripa et al., 2020; Pi et al., 2021). We slid the EEG data recorded in the experiment with a window size of 4 s to calculate the power of the five frequency bands corresponding to each window. By this means, we obtained the relative power changes of each participant in the five frequency bands during the learning process as well as the average power of all participants. For the same sliding windows, we calculated the approximate entropy value to present the pattern repeatability of the EEG time series. A larger entropy indicates less predictability.

Meanwhile, we calculated the average pupil dilation data for each trial stimulus. To synchronize with EEG features, we calculated the average of the eye tracking features in the corresponding window size (i.e., 4 s).

## Results

One-way analysis of variance (ANOVA) and Mann-Whitney *U* tests were used to make comparisons across the two groups. One-way ANOVA was used on the dependent variables that met the normality assumption to conduct an ANOVA. The Mann-Whitney *U* test was used as a non-parametric test of equivalence on dependent variables whose skewness and kurtosis values indicated that they did not meet the assumption of normality (Fritz et al., 2012; Legesse et al., 2020). The Mann-Whitney *U* test is most sensitive to changes in the medium.

Effect sizes were measured by $\eta_{p}^{2}$ for the one-way ANOVA, with $\eta_{p}^{2}$ = 0.01 considered a small effect, 0.06 a medium effect, and 0.14 a large effect (Cohen, 1988).

Effect size were estimated by Mann-Whitney *U* tests, the *z* value can be used to calculate an effect size, such as the r proposed by Cohen (1988). Cohen’s guidelines for r are that a large effect is 0.5, a medium effect is 0.3, and a small effect is 0.1 (Coolican, 2009).

### Control Variables

**FIGURE 3:**
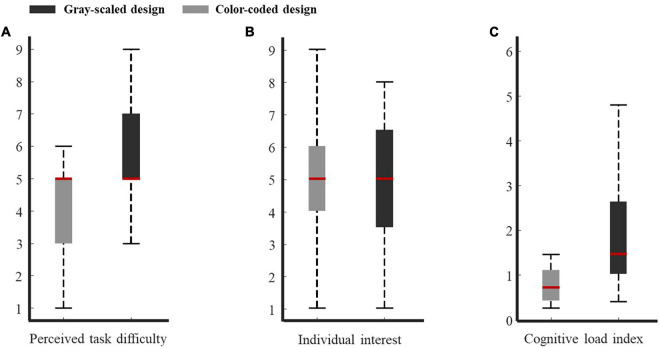
Boxplot data for participants in two conditions. **(A)** Perceived task difficulty. **(B)** Individual interest. **(C)** Cognitive load.

The first step in the analysis was to determine whether there were differences in prior knowledge and individual interest between the two groups. The descriptive statistics of control variables under the two conditions are presented in Table 1. Figure 3A showed boxplot of subjective ratings of individual interest in the two conditions. For each variable (i.e., prior knowledge and individual interest), a one-way ANOVA was conducted with the group as the between-subjects factor. The results revealed no main effect for either control variable. For prior knowledge, *F*(1,38) = 0.18, *p* = 0.67; for individual interest, *F*(1,38) = 0.11, *p* = 0.75. The results indicate that there was no difference between the two learning conditions in terms of prior knowledge and individual interest.

**TABLE 1:** Means and standard deviations for control variables.

	Color-coded group (*N* = 19)		Gray-scaled group (*N* = 19)	
Dependent variable	*M*	*SD*	*M*	*SD*
Prior knowledge	80.20	2.82	80.10	2.14
Individual interest	4.95	2.04	4.74	2.08

### Learning Performance

As illustrated in Table 2, the participants who studied the color-coded video lecture had higher scores on the performance test than those who studied the grayscale video lecture. A one-way ANOVA revealed that the learning performance across the two groups was statistically significant, with *F*(1,38) = 5.65, *p* = 0.028, $\eta_{p}^{2}$ = 0.23, indicating that the color-coded group performed better than the grayscale group in the post-test. The effect of the material format on learning performance was found to be significant with a large effect size. This result supports our hypothesis.

**TABLE 2:** Means and standard deviations of learning performance and perceived task difficulty.

	Color-coded group (*N* = 19)		Gray-scaled group (*N* = 19)	
Dependent variable	*M*	*SD*	*M*	*SD*
Learning performance	5.09	2.07	3.10	1.73
Perceived task difficulty	4.28	1.23	5.71	1.76

### Subjective Ratings of Perceived Task Difficulty

Figure 3B showed boxplot of subjective ratings of perceived task difficulty in the two conditions. The one-way ANOVA of task difficulty scores revealed a main effect for color-coded design, with *F*(1,38) = 7.83, *p* = 0.009, $\eta_{p}^{2}$ = 0.19. As illustrated in Table 2, learners who viewed grayscale video lectures rated the difficulty of the learning material to be higher than those who received color-coded video lectures. The results revealed that the color-coded design reduced the perceived difficulty of the learning task.

### Physiological Measures of Cognitive Processing

#### Eye-Tracking Data Analysis

The descriptive statistics of all eye-tracking variables in the two conditions are presented in Table 3. A one-way ANOVA showed that there was a significant main effect of color coding design (*F*(1,37) = 8.28, *p* = 0.008, $\eta_{p}^{2}$ = 0.24; *F*(1,37) = 5.19, *p* = 0.031, $\eta_{p}^{2}$ = 0.13) on left and right pupil diameters. The subjects who were given the color-coded material had a smaller left pupil diameter than those who were given the grayscale material. Accordingly, the right pupil diameter was significantly larger in the grayscale condition than in the color-coded condition.

**TABLE 3:** Means and standard deviations of eye-tracking variables.

	Color-coded group (*N* = 19)		Gray-scaled group (*N* = 19)	
Dependent variable	*M*	*SD*	*M*	*SD*
Pupil diameter left	3.29	0.35	3.71	0.42
Pupil diameter right	3.37	0.31	3.72	0.46
Fixation duration	218.12	103.23	314.00	137.50

One-way ANOVA also revealed a significant difference in gaze event duration on color-coded design across experimental conditions, with *F*(1,37) = 4.35, *p* = 0.047, $\eta_{p}^{2}$ = 0.143. Participants who viewed video lectures with grayscale design had longer fixation durations than those who viewed video lectures with color-coded design.

#### Electroencephalography Data Analysis

##### Electroencephalography Power

The descriptive statistics of all variables of EEG power under the two conditions are presented in Table 4.

**TABLE 4:** Means and standard deviations of EEG power variables.

	Color-coded group (*N* = 19)		Gray-scaled group (*N* = 19)	
Dependent variable	*M*	*SD*	*M*	*SD*
Delta	38.00	15.85	35.26	18.9
Theta	16.12	5.83	10.70	5.08
Alpha	16.37	8.60	11.72	4.36
Beta	26.46	13.02	38.11	18.92
Gamma	3.11	1.90	4.58	2.39

One-way ANOVA was conducted with the between-subjects factors of the color-coded design condition and delta power, theta power, alpha power, beta power, and gamma power as the dependent variables. The results are shown in Table 4.

The one-way ANOVA revealed significant main effects of color coding (*F*(1,37) = 9.36, *p* = 0.004, $\eta_{p}^{2}$ = 0.206; *F*(1,37) = 4.43, *p* = 0.042, $\eta_{p}^{2}$ = 0.110; *F*(1,37) = 4.89, *p* = 0.034, $\eta_{p}^{2}$ = 0.119; *F*(1,37) = 4.40, *p* = 0.043, $\eta_{p}^{2}$ = 0.109) on theta, alpha, beta and gamma power. However, there was no difference in delta power between the two learning conditions.

As shown in Table 4, the participants had higher theta and alpha power when they were shown a color-coded video lecture, and in turn had higher beta power when they were shown a grayscale video lecture.

**FIGURE 4:**
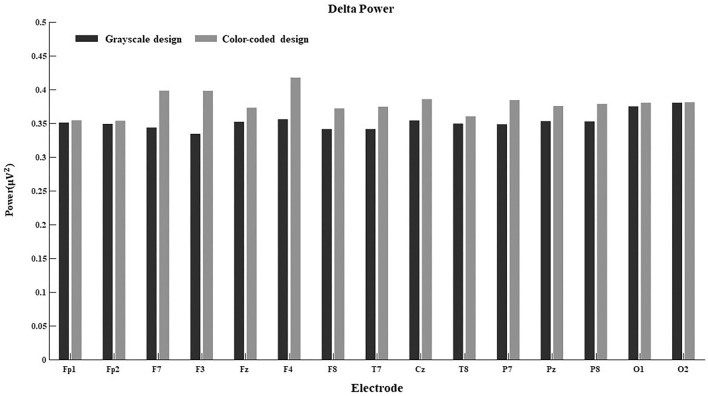
Delta power for the 15 electrodes in the grayscale and color-coded conditions while watching video lectures.

**FIGURE 5:**
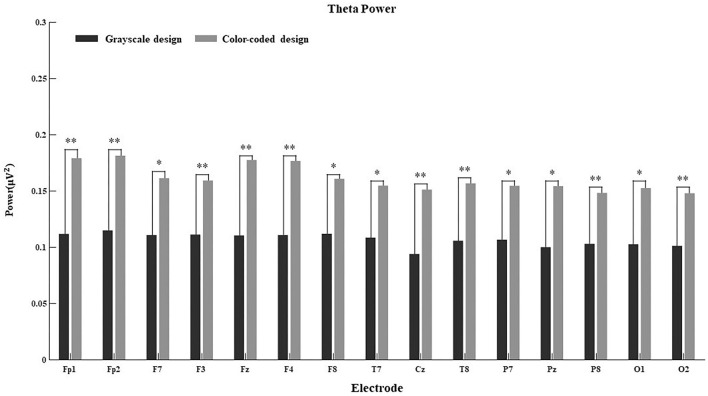
Theta power for the 15 electrodes in the grayscale and color-coded conditions while watching video lectures (*p < 0.05, **p < 0.01).

**FIGURE 6:**
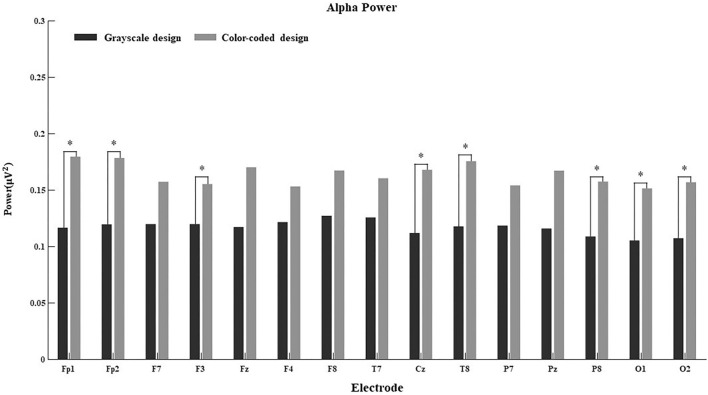
Alpha power for the 15 electrodes in the grayscale and color-coded conditions while watching video lectures (*p < 0.05).

**FIGURE 7:**
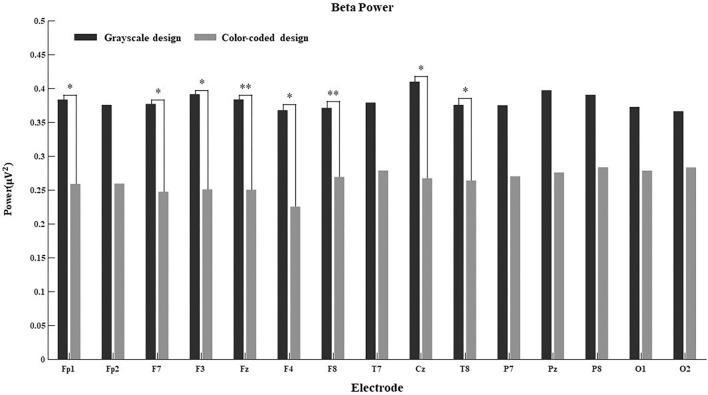
Beta power for the 15 electrodes in the grayscale and color-coded conditions while watching video lectures (*p < 0.05, **p < 0.01).

**FIGURE 8:**
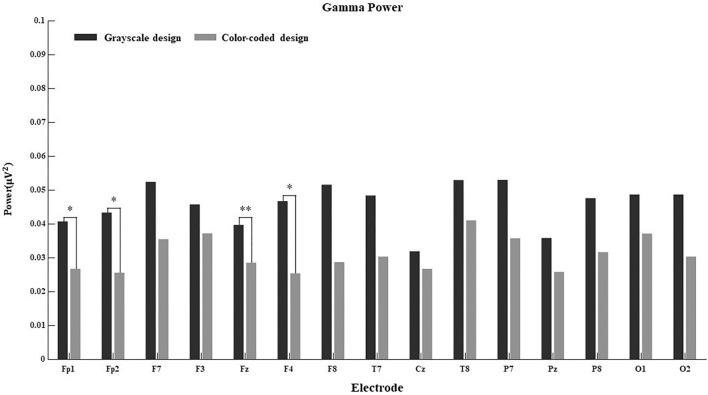
Gamma power for the 15 electrodes in the grayscale and color-coded conditions while watching video lectures (*p < 0.05, **p < 0.01).

Since the connection between EEG power and cognitive processes is more obvious in some regions than others, we further calculated the main effect of different electrodes on delta power, theta power, alpha power, beta power, and gamma power in both conditions. The results are shown in Figures 4–8.

There was no difference in the 15 electrodes on delta power across the two learning conditions (Figure 4). The participants had significantly higher theta power when learning through color-coded video lectures than through grayscale ones in all electrode regions (see Figure 5). In the Fp1, Fp2, F3, T8, Cz, P8, O1, and O2 regions, the participants had significantly higher alpha power when learning through color-coded video lectures than through grayscale ones (see Figure 6). There was a significant difference in beta power in the Fp1, F3, F7, F8, Fz, F4, T8, and Cz regions across the two learning conditions (see Figure 7). For gamma power, the Fp1, Fp2, Fz, and F4 regions showed significant differences (see Figure 8).

We chose the regions that had significant differences and adopted β/(θ + α) to evaluate the cognitive load. β/(θ + α) has proven to be very effective in quantifying the state of mental workload (Berka et al., 2007). The subjects who received the grayscale learning material had a higher cognitive load (*M* = 2.05, SD = 1.56) than those who received the color-coded learning material (*M* = 0.96, SD = 0.87). Figure 3C showed boxplot of cognitive load in the two conditions. The Mann-Whitney *U* test likewise revealed a significant difference across the two study conditions in cognitive load (*U* = 85, *p* = 0.005, *r* = 0.45). The effect of the material format on cognitive load was found to be significant, with a large effect size. This outcome indicates that the participants who viewed grayscale video lectures required more mental processing.

##### Spectral Entropy

The descriptive statistics of all variables of spectral entropy under the two conditions are presented in Table 5.

**TABLE 5:** Means and standard deviations of spectral entropy variables.

	Color-coded group (*N* = 19)		Gray-scaled group (*N* = 19)	
Dependent variable	*M*	*SD*	*M*	*SD*
Approximate entropy	1.19	0.093	1.44	0.10
Sample entropy	0.60	0.43	0.66	0.044
Wavelet entropy	2.13	0.019	2.14	0.018

One-way ANOVA revealed statistically significant differences across the experimental conditions on approximate entropy (*F*(1,37) = 624.03, *p* < 0.001, $\eta_{p}^{2}$ = 0.617), sample entropy (*F*(1,37) = 191.58, *p* < 0.001, $\eta_{p}^{2}$ = 0.331), and wavelet entropy (*F*(1,37) = 27.31, *p* < 0.001, $\eta_{p}^{2}$ = 0.069). The results indicated that learners who received the grayscale learning material had higher spectral entropy than those who received the color-coded learning material. The results suggest that compared to participants who watched color-coded video lectures, the participants who watched grayscale video lectures had a heavier mental load.

## Discussion

The main purpose of the present study was to investigate the impact of color coding on programming learning in multimedia learning by measuring learning performance and cognitive processing using a combination of eye tracking and EEG data. The results indicated that the participants who studied the learning material with the color-coded design had higher learning performance than those who studied the learning material with the grayscale design. Our findings are in line with those of a previous study on the color coding effect (Ozcelik et al., 2009; Stark et al., 2018). Hence, we confirmed that color coding can promote programming learning in multimedia learning.

Color-coded materials minimize unnecessary search processes used to correlate verbal information, which may help improve performance. Our results show that the fixation duration on color-coded material was shorter than that on grayscale material. This indicates that participants are able to search for associated elements between visual and verbal information more quickly in the color-coded format than in the grayscale format. Utilizing the same color to relate elements in a slide could guide learners to focus on the salient and relevant information (Navalpakkam et al., 2005). This is because salient stimuli can automatically capture attention and affect the perceptual choice of subjects when processing information, so as to shorten the information search time and release more cognitive resources for information integration and material organization (Mayer, 2005b; Schnotz, 2014).

The current results are in line with the temporal contiguity effect in multimedia learning (Mayer, 2014). Our data provide evidence that color coding helps to form an association between verbal and program code representations because the items processed timely are related to each other (Raaijmakers and Shiffrin, 1981; Kahana, 1996). Color coding facilitates the identification of corresponding visual and verbal elements, and improves learning performance through the temporal proximity of relevant information (Ginns, 2006).

When learners are able to easily ascertain relevant information and integrate verbal and visual information, they can engage more deeply in the cognitive processing required for meaningful learning (Mayer and Chandler, 2001; Mayer and Moreno, 2003; Seufert, 2003). The present study observed higher theta and alpha powers in the color-coded learning conditions than in the grayscale learning conditions. The theta band is related to the working memory. The increases in theta oscillations are related to high working memory activity as well as successful memory encoding (Miller et al., 2018) and retrieval (Solomon et al., 2019; Pi et al., 2021). Therefore, deep processing of learning material in working memory can successfully encode memory (Khader et al., 2010; Lin et al., 2017), and working memory maintenance can forecast long-term memory (Hsieh and Ranganath, 2014). The learners who successfully memorized learning materials showed higher theta power than those who did not (Khader et al., 2010). Neural oscillations in the alpha band are related to an increase in internal attention-demanding cognitive processing (Klimesch, 2012; Fink and Benedek, 2014). Our study suggests that the color-coded group had a greater engagement of working memory and attention than the grayscale group, allowing information to be processed at a deeper level and thus resulting in better learning performance (Pi et al., 2021).

Fixation durations within the same activity can also reflect cognitive processing and cognitive load (Kruger and Doherty, 2016); a longer fixation duration suggests an increased cognitive load. Furthermore, the higher the difficulty of text comprehension and the greater the memory load during learning, the greater the pupil enlargement (Kahneman and Beatty, 1966, 1967). The results of the present study showed that the participants who viewed grayscale video lectures had longer fixation durations and greater pupil size than those who viewed color-coded video lectures. This indicated that the participants under the grayscale learning condition had a higher cognitive load than those under the color-coded condition. These results are consistent with the Cognitive Load Theory (Sweller, 1988, 1994) and the Cognitive Theory of Multimedia Learning (Mayer, 2005b). In the process of multimedia learning, learners need to psychologically integrate information transmitted through external representations into a coherent psychological representation. If learners utilize their limited cognitive resources to search the text when they hear new words in the narration, the visual scanning activity will produce extraneous cognitive processing (Paas and Sweller, 2014; Wang et al., 2020). Prior research has shown that learners usually need instructional support to identify correspondences and establish connections between them (e.g., Seufert, 2003; Seufert and Brünken, 2006). As a mode of signaling, color coding could guide learners’ attention toward essential material and thereby eliminate the need to process irrelevant material. Color coding highlights the organization of the essential material to guide learners’ cognitive processing. Specifically, it reduces extraneous mental processing demands to allow people to learn more deeply, which in turn leads to improved learning performance.

Using color coding could reduce the search for reference in the text and improve split-attention effects, resulting in lower perceived task difficulty (Ayres and Sweller, 2014; Xie et al., 2016), which is a reliable measure of extraneous cognitive load (Kalyuga et al., 2000). Subjective ratings of task difficulty increase as the extraneous cognitive load increases (Paas and van Merrienboer, 1993; Paas et al., 1994). The study results indicate that the participants in the grayscale group had more extraneous cognitive load than the participants in the color-coded group. Extraneous cognitive load is caused by the inappropriate presentation of the learning material (Sweller, 2004). When the extraneous cognitive load is increased, the germane cognitive load is reduced and learning is lessened. Learners use limited working memory resources to deal with the extraneous factors imposed by the teaching process rather than the intrinsic material. The more working memory resources are needed to deal with the extraneous cognitive load, the less resources are available to deal with the intrinsic cognitive load, which in turn reduces learning (Sweller, 2010). Meanwhile, color coding is an effective multimedia design method that minimizes extraneous overload (van Gog, 2014); it helps people study more deeply in multimedia learning because the added cues emphasize the organization of the essential material. The results indicated that the index β/(θ + α) could measure the extraneous cognitive load during the learning process.

The results also provide evidence for the emotional design hypothesis (e.g., Mayer and Estrella, 2014; Plass et al., 2014). Making essential elements visually appealing (i.e., colored) directs cognitive processing (i.e., selecting) in the learning process by guiding attention, maintaining cognitive processing, and helping learners better understand the essential material (i.e., organizing and integrating) – thereby leading to better learning (Pekrun, 2006).

## Conclusion

This study provides evidence for the benefits of using color coding in programming learning during multimedia learning. We utilized multimodal physiological measures to access process information for a detailed analysis of the programming learning process using color-coded design and grayscale design in multimedia learning materials. We likewise combined EEG frequency band power data and eye tracking data to measure mental processing. The results demonstrate that color coding guides learners in finding corresponding visual and verbal information and paying attention to critical information for meaningful learning. The participants under the color-coded condition had a lower cognitive load, more positive learning experience, and better learning performance than those under the grayscale condition.

The results of the present study suggest that using color coding to highlight the format of program code in video lectures for programming learning can improve learning performance. Learning material might be better designed to help learners select relevant information and integrate verbal and visual representations so as to enhance learning (Mayer and Fiorelle, 2014). The conclusions of this study can also be extended to other courses.

## Data Availability Statement

The datasets presented in this study can be found in online repositories. The names of the repository/repositories and accession number(s) can be found below: https://github.com/zeron21/Multimodal_Data.git.

## Ethics Statement

The studies involving human participants were reviewed and approved by the Ethics Committee of the School of Information and Electronic Engineering, Zhejiang University of Science and Technology. The participants provided their written informed consent to participate in this study.

## Author Contributions

All authors listed have made a substantial, direct, and intellectual contribution to the work, and approved it for publication.

## Conflict of Interest

The authors declare that the research was conducted in the absence of any commercial or financial relationships that could be construed as a potential conflict of interest.

## Publisher’s Note

All claims expressed in this article are solely those of the authors and do not necessarily represent those of their affiliated organizations, or those of the publisher, the editors and the reviewers. Any product that may be evaluated in this article, or claim that may be made by its manufacturer, is not guaranteed or endorsed by the publisher.
